# P16 Overuse of antibiotics during the first year of the COVID-19 pandemic in Poland

**DOI:** 10.1093/jacamr/dlac133.020

**Published:** 2023-01-19

**Authors:** Izabella Owsianka, Mateusz Gajda, Paweł Krzyściak, Barbara Gryglewska, Anna Różańska, Jadwiga Wójkowska-Mach

**Affiliations:** Chair of Microbiology, Medical Faculty, Jagiellonian University Medical College, Krakow, Poland; Chair of Microbiology, Medical Faculty, Jagiellonian University Medical College, Krakow, Poland; Chair of Microbiology, Medical Faculty, Jagiellonian University Medical College, Krakow, Poland; Department of Internal Medicine and Gerontology, Medical College, Jagiellonian University, Kraków, Poland; Chair of Microbiology, Medical Faculty, Jagiellonian University Medical College, Krakow, Poland; Chair of Microbiology, Medical Faculty, Jagiellonian University Medical College, Krakow, Poland

## Abstract

**Background:**

It was a retrospective study conducted at the University Hospital in Kraków in Poland (UHC). The objectives of the study identify the trends of the antimicrobial consumption profile from February 2020 to February 2021, the first year of the COVID-19 pandemic in Poland. The hospital records data have been analysed to identify factors that were associated with antimicrobial consumption and illustrate how the situation evolved over time. The secondary objective is to identify the determinants of antimicrobial use.

**Methods:**

The analysis was carried out on data based on electronically registered health care data from the UHC and hospital pharmacy. We included 250 adult patients, divided into 5 groups, and hospitalized with confirmed SARS-CoV-2 infection. The study began on 13 February 2020, when the patient with a confirmed hospital-acquired (HA)-COVID-19 in March was admitted to UHC. Next, we enrolled 49 consecutive patients and then, consecutive 50 patients admitted to the UHK on 13 May 2020, 13 August 2020, 13 November 2020 and 13 February 2021. Patients with HA-COVID-19 were eligible for inclusion in this study.

**Results:**

In total, 178 patients received antimicrobials (ABX), 71.2% of all and the incidence rate of laboratory confirmed healthcare-associated infection (LC-HAI) was 20%. No patients had laboratory-confirmed bacterial co-infection at admission. The severity of COVID-19 was mild in 102 (40.8%), moderate in 92 (36.8%), and severe in 56 (22.4%) patients. Forty-three (17.2%) patients were hospitalized in an ICU, and 97.7% of them were treated with antimicrobials. The length of stay was greater among patients receiving ABX. The percentage of patients receiving antibiotic therapy changed throughout the covered time periods. The lowest proportion was recorded in May 2020 (48.0%), whereas the highest was observed in November 2021 and February 2021 (84.0% both). Systemic glucocorticoids (GCS) were used in 117 patients, 46.8% of all, significantly more frequently in patients with ABXs *P*<0.001. Patients with severe COVID-19 had the highest antimicrobial consumption values, the median DDD in this group was 20.92, *P*<0.001. Patients admitted at the beginning of the pandemic (February, May 2020) had significantly higher median DDD values, respectively 25.3 and 16.0 compared with those admitted in later months of the pandemic (August, November 2020; February 2021), 11.0, 11.0 and 11.2.

**Conclusions:**

The results show that despite COVID-19 being a viral disease, the use of antimicrobials has been common in infected patients, especially at the beginning of the pandemic. The observed incidence of LC-HAI did not change during 12 months of follow-up and was very high, despite the fact that only 1/5 of the patients were hospitalized in the ICU.

